# Towards disease-oriented dosing of rapamycin for longevity: does aging exist or only age-related diseases?

**DOI:** 10.18632/aging.204920

**Published:** 2023-07-20

**Authors:** Mikhail V. Blagosklonny

**Affiliations:** 1 Roswell Park Comprehensive Cancer Center, Buffalo, NY 14263, USA

**Keywords:** mTOR, hyperfunction, lifespan, health span, cancer, Alzheimer’s disease

## Abstract

Both individuals taking rapamycin, an anti-aging drug, and those not taking it will ultimately succumb to age-related diseases. However, the former, if administered disease-oriented dosages for a long time, may experience a delayed onset of such diseases and live longer. The goal is to delay a particular disease that is expected to be life-limiting in a particular person. Age-related diseases, quasi-programmed during development, progress at varying rates in different individuals. Rapamycin is a prophylactic anti-aging drug that decelerates early development of age-related diseases. I further discuss hyperfunction theory of quasi-programmed diseases, which challenges the need for the traditional concept of aging itself.

## Prologue

Rapamycin (Sirolimus) is the only one drug that consistently extends life span in countless animal studies in all species tested [1–19] and see for ref: [20–22].

These results had been predicted by hyperfunction theory of quasi-programmed aging, which presents rapamycin as a universal anti-aging drug that suppresses cellular senescence and organismal aging, and thus decelerates development of age-related diseases, deadly manifestations of aging [23]. Aging is driven in part by hyper functional growth-promoting pathways such as mTOR. By slowing aging, rapamycin delays age-related diseases [23–25].

I emphasize that aging is not programmed but, in contrast, quasi-programmed. Quasi means pseudo; seemingly; apparently but not really. Some scientists deliberately represent hyperfunction theory as theory of programmed aging. It’s the opposite. Quasi-program is a continuation of a real program. Quasi-program has no intent, no purpose and it is always harmful. These quasi-programs are diseases, potentially deadly (e.g., hypertension, prostate cancer) or not (e.g., presbyopia, wrinkles). Quasi-programs provide no benefit. As previously discussed, these quasi-programmed are driven by hyperfunctional cells, signaling pathways and systems (see for references [26–28]. Without discussing these topics here, it is noteworthy that SASP (hyper-secretion) and pro-inflammation are typical hyperfunctions [29, 30].

## Rapamycin for life-limiting disease/condition

Currently, doses and schedules of rapamycin for longevity are based on the wrong objective: to avoid side effects. Nothing else. The doses of all other drugs are chosen to achieve therapeutic goals. For aspirin’s example, rheumatoid arthritis patients received high-dose aspirin (3600 mg/day) daily [31], while for prevention of cardiovascular diseases we use low-dose aspirin (81 mg). Depending on the pathology, aspirin usage can be intermittent or continuous [32, 33]. Side effects avoidance is a secondary goal, even though side effects may be deadly.

Side effects of rapamycin are not remarkable at all. They even less dangerous than the side effects of most other drugs [34, 35]. Since 1999, millions of patients with serious illnesses tolerated rapamycin well. Continuous (everyday) even high doses were studied successfully in patients [36]. A failed suicide attempt (103 tablets or 103 mg) caused no effects except elevated blood lipids [37]. In some studies, side effects were higher in the placebo group than in the rapamycin-treated group [38].

However, what’s most significant is that many so-called “side” effects of rapamycin can actually be considered therapeutic effects themselves or indicative markers of therapeutic effects. For instance, mild anemia may be viewed as a marker for the cytostatic effect, which is critical in inhibiting tumor progression. This serves as an example of the latter. As for the former, rapamycin decreases levels of blood insulin, causing glucose intolerance. Most metabolic “side” effects of rapamycin are therapeutic and associated with increased lifespan in animals. In humans, metabolic effects of rapamycin may depend on the diet [39].

Based on “side” and therapeutic effect-avoidance, the most popular schedule of rapamycin for longevity is 5–7 mg once a week. The schedule is well tolerated and lacks side effects except of rare mouth sores [40]. It is based on the assumption that the intermittent schedule has fewer side effects than everyday doses. But this never was compared. For example, treatment with 1 mg rapamycin every day lacks side effects in healthy elderly [41]. So, both schedules have negligible side effects. But are they equally effective for life extension? We do not know.

In 2006, when the hyperfunction theory of aging was published [23], I initially envisioned that rapamycin should be administrated at continuous (everyday), low doses (0.5 mg/day) to prevent age-related diseases. Later, experiments in mice suggested that low doses are suboptimal [42].

In mice, the higher the dose, the longer lifespan [6, 12, 35, 43]. Therefore, in humans, the highest dose that does not yet cause unacceptable side effects (maximal tolerated dose) may be optimal for longevity. If (unacceptable) side effects develop, the dose should be decreased. In other words, anti-aging doses are maximal doses without side effects in a particular person [34]. Then anti-aging doses are individual and side-effect-free by definition.

In 2006, I believed that optimal treatment is low dose everyday (not intermittent) [23]. By 2008, I recognized that this not the only one way to use rapamycin. In theory, intermittent treatment may rejuvenate stem and wound healing cells [44].

Since 2009, data accumulated in mice that not only everyday treatment [2, 3, 6–9], but also various types of intermittent and even transient treatments [1, 4, 5, 10–14] with rapamycin successfully extended life span in mice.

In theory, high intermittent dose of rapamycin may produce a high peak level to ensure that even rapamycin-resistant cells will be targeted. A high peak concentration may affect neurons, protected by the blood brain barrier, and stem cells in their niches. A high single dose of rapamycin was shown to maintain lower body weight by shifting the set point long-term in rats [45].

While intermittent therapy offers some benefits, it may also pose challenges due to drug-free periods potentially causing mTOR over-activation, theoretically leading to harm or accelerated pathology. The ideal dosage and schedule should therefore be individualized based on age, gender, and each person’s unique spectrum of pre-diseases.

Nevertheless, extremely high intermittent concentrations of blood rapamycin, which would be unattainable in humans, can prove to be counterproductive in female mice [12]. One study demonstrated that high doses of rapamycin were less effective in preventing cancer in prostate epithelium-specific Pten-knockout mice when compared to low doses [46].

Dosing and scheduling of rapamycin may vary, largely depending on the early signs of the pathology in question, and therefore, the specific cell type that is being targeted.

It’s essential to note that no one dies from aging itself, but rather from diseases that are age-related. Even all centenarians die from age-related diseases [47]. We will further explore this crucial point in the subsequent section, highlighting that aging doesn’t exist independently from pathology. Rather, it’s an abstract concept that collectively represents all age-related pathologies.

Figuratively, aging (a continuation of development) drives all age-related diseases. By targeting “aging”, we may delay or prevent age-related diseases [23, 24]. This approach was later named the geroscience hypothesis. Now, I consider this as oversimplification. Aging is merely a set, consistent of individual age-related diseases. According to upgraded hyperfunction theory, quasi-programmed aging is replaced by quasi-programmed diseases [28]. These diseases, not a virtual aging, is a continuation of developmental programs, so they are quasi-programmed and hyprerfunctional. Quasi-programmed diseases are, in part, mTOR-dependent, a continuation of mTOR-driven cellular growth [28].

Aging has a mathematical meaning, as a sum of all age-related diseases and other quasi-programs, but not precise biological meaning. Aging is defined as exponential increase of mortality with age, exactly because it consists of quasi-programmed diseases, whose incidence and mortality increases exponentially with age.

And when a complication of either one or several interacting age-related diseases (ARDs) kills a human, prevention of the other diseases cannot extend lifespan. Lifespan and healthspan are often determined by a life-limiting disease in a particular individual and doses and schedules of rapamycin should be individual.

The goal of rapamycin treatment is to prevent particular life-limiting age-related diseases that would kill a particular person.

The key word is “life-limiting”. To extend lifespan, the treatment must delay the life-limiting disease. In medical science, it’s simple. If a patient is dying from cancer, it is cancer (a life-limiting disease in this patient) that is treated, rather than a disease like Alzheimer’s that is not yet present.

This principle applies in geroscience as well. For instance, a healthy aging smoker with a family history of cancer should be prescribed rapamycin specifically to delay lung cancer, not Alzheimer’s disease. Conversely, an aging person with a genetic predisposition to Alzheimer’s should receive a regimen of rapamycin tailored to thwart that disease. Though rapamycin may prevent both cancer [48] and Alzheimer’s disease [49], optimal doses and schedules differ between the diseases.

Theoretically, high intermittent doses could target brain cells despite the blood-brain barrier (BBB). This process is intricate, as rapamycin’s effects on the BBB, via endothelial cells, need to be factored in when designing dosages.

Therefore, the doses and schedules of interventions like rapamycin should be tailored to the specific cell types. Each disease’s mechanism involves its unique set of cells. For instance, development of atherosclerosis involves various cells such as arterial smooth muscle cells, endothelial cells, macrophages, blood platelets, hepatocytes and fat cells and others.

Several cell types are involved in diseases and conditions such as hair loss, prostate enlargement, menopause, atherosclerosis, Parkinson’s and so on [28]. Different types of cells may differ in their sensitivity to rapamycin.

Thus, optimal doses and schedules of rapamycin are different, depending on the life-limiting pathology expected in an individual. The goal is to prevent diseases that would kill a particular person.

## Does aging truly exist?

The preceding chapter posited that anti-aging treatment must be disease-oriented, a perspective I echo by proposing a disease-centric theory of aging. In essence, aging becomes an unnecessary concept under scrutiny. When examined closely, what we perceive as “aging” disintegrates, replaced instead by a spectrum of age-related diseases (ARDs) and conditions such as grey hair. It is important to note that for succinctness, “ARD” will henceforth encompass all age-related diseases, pre-diseases, and age-related benign conditions [50]. In this context, the term “ARD” will strictly refer to age-related quasi-programmed diseases. Given this premise, what are the prevailing perspectives on the interplay between aging and ARDs?

**Figure 1:**
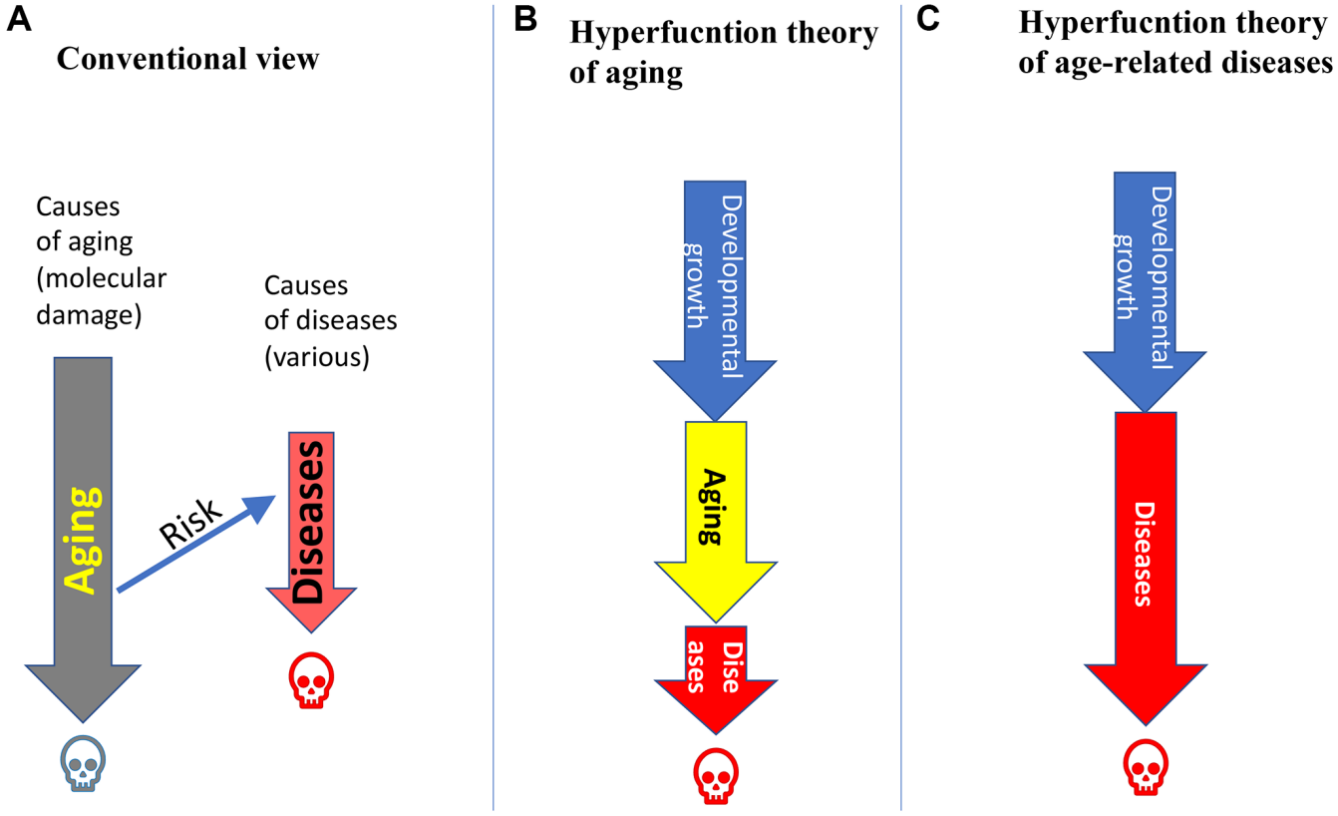
**Relations between aging and age-related diseases (ARDs).** (**A**) Aging is caused by accumulation of molecular damage. ARDs are caused by external causes. Aging is a just a risk factor for ARDs. Humans die from aging (grey skull), unless they die earlier from ARDs (red skull). (**B**) Hyperfunction theory of quasi-programmed aging. Aging is a continuation of developmental growth, driven in part growth-promoting pathways such as mTOR and MAPK. Aging is manifested by ARDs, which kill a person. (**C**) Hyperfunction theory of quasi-programmed ARDs. ARDs are a continuation of developmental growth, driven in part by growth-promoting pathways such mTOR and MAPK.

According to a dominating notion, aging is caused by accumulation of molecular damages, leading to functional decline and death. Age-related diseases (ARDs) are caused by other causes, such as unhealthy lifestyle and “genetics” (Figure 1A). Accordingly, aging is just a risk factor for diseases (but this explains little. Why is it a risk factor?) but not a cause. (For example, hypertension is not caused by accumulation of molecular damages). With a healthy lifestyle, aging will cause a “healthy” death (I slightly inflate). Otherwise, a person will die prematurely from age-related diseases (Figure 1A).

According to hyperfunction theory of quasi-programmed aging [23], aging is a continuation of developmental growth programs. When developmental growth is completed, the mTOR growth-promoting pathway drives aging instead of growth. Its activity is optimal for growth but higher than necessary post-developmentally. Hyper functional signaling renders cells hyper functional, driving age-related pre-diseases and diseases [23, 25, 28]. Age-related diseases (ARDs) in turn lead to secondary loss of functions and organ failure (late manifestations of aging) [23]. Hyperfunction theory explains why quasi-programmed aging is life-limiting, whereas accumulation of molecular damage is not life-limiting [23, 26].

According to hyperfunction theory, aging is a common driving cause of all ARDs, not just a risk factor (Figure 1B). These diseases are obligatory manifestations of aging. Diseases, not aging per se, cause death in animals from humans to *C. elegans* [51–53]. Aging and, therefore, ARDs, such as hypertension, are not caused by molecular damage. (Note: Hypertension is a continuation of a developmental increase of blood pressure started from birth).

The hyperfunction theory of aging is a convenient approximation. Here, I attempt a major revision of hyperfunction theory. It is a hyperfunction theory of quasi-programmed diseases.

At first glance, aging behaves as a complex disease and can be treated as a disease by potential anti-aging drugs, but such treatment should be individualized (see section 1).

Under magnification, aging is not a disease but a set of all diseases, in mathematical sense, and a set of diseases is not a disease. In analogy, a zoo consists of animals, but a zoo is not an animal [28].

Aging is correctly defined as an exponential (at least in humans) increase of the probability of death with age, because aging consists of age-related diseases (ARDs) that kill exponentially with age. Although an exponential increase is not perfect for each disease, as a sum, it gives a perfect exponential curve.

Aging is a useful abstraction, which mathematically behaves as an age-related disease but does not exist as independent entity. It is a sum of all age-related pre-diseases, age-related diseases and conditions. Fragility, gray hair, atherosclerosis and numerous condition and diseases, all together are called aging. But aging does not exist without these diseases.

Aging can be likened to a bouquet of different flowers, where each flower represents a disease and the bouquet represents aging. Just as a bouquet is simply a collection of flowers, aging is a collection of age-related diseases, which are quasi-programmed in development. Each disease (or flower) exists independently, but the concept of aging (the bouquet) is formed by these diseases combined. To understand the aging process (the decay of the bouquet), one must study the individual diseases (the state of each flower). For instance, if tulips in the bouquet begin to rot first, they could be removed, akin to treating a life-limiting disease.

Aging does not exist as a real, solo, undivided, uniform and homogenous process. It is elusive and illusive. It consists of multiple processes: age-related diseases, quasi-programmed in development.

**Figure 2:**
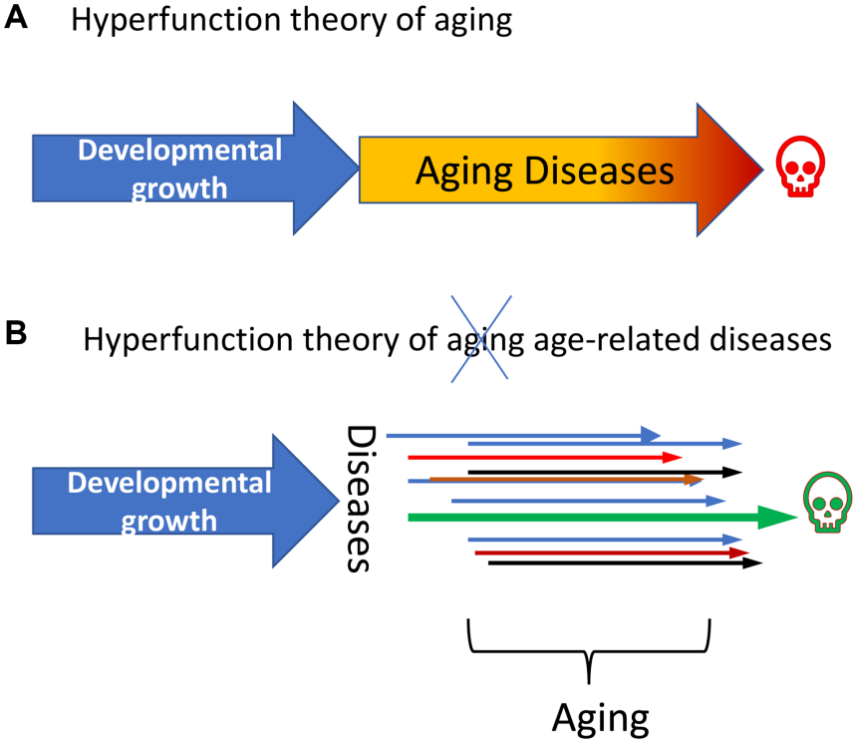
**Age-related diseases (ARDs) are quasi-programed in development.** (**A**) Hyperfunction theory of quasi -programmed aging. Aging is a continuation of developmental growth. (Note: Aging is not programmed, it is quasi-programmed.) ARDs are manifestations of aging. (**B**) Hyperfunction theory of quasi -programmed ARDs. Each individual ARD (shown as colored arrows) is a continuation of developmental growth. Life-limiting disease (green arrow) terminates life. Aging is an abstraction representing a sum of all diseases and conditions.

Let us compare two versions of hyperfunction theory of: (i) quasi-programmed aging and (ii) quasi-programmed diseases (Figure 2).

First, aging is a quasi-program, it is a continuation of developmental growth and reproductive programs. Aging drives age-related diseases (Figure 2A). Genetic variability and environmental hazards also contribute (see [28]). Diseases terminate lifespan.

Second, age-related diseases are quasi-programs, they are continuations of developmental growth and reproductive programs. Developmental programs directly drive age-related diseases (no intermittent “virtual aging”) (Figure 2B). Genetic variability and environmental hazards also contribute (see [28]). Diseases terminate lifespan.

Under high magnification “aging” looks multi-faceted, analogous to individual grains (diseases) seen under a microscope (Figure 2). Aging (a set of diseases) is driven by but multiple quasi-programs, offshoots of developmental programs that are not deactivated. Although mTOR-driven cellular hypertrophy, hyperplasia, hyperfunctions are involved in prostate enlargement and atherosclerosis, these two quasi-programs are different, because different cell types participate in them (see [28]). Age-related diseases (ARDs) are only partially quasi-programmed [28]), because both environmental and genetic factors contribute to them. Not all quasi-programs are TOR-dependent. For instance, the epigenetic clock, a measure of biological aging based on DNA methylation levels, appears to be mTOR-independent.

The essence of aging seems difficult to pinpoint because it has mathematical, rather than biological, meaning. It is a mathematical set of all diseases and conditions. These processes have biological meaning, an aimless and unintended continuation of developmental growth programs that were not switched off upon their completion. These quasi-programs are associated with cellular and systemic hyperfunctions therefore eventually causing organ damage and secondary loss of functions. Fragility is manifestations of end-stage pathologies with secondary loss of function. To understand a sum of quasi-programs (aging), we need to study early-life hyperfunctions.

## In conclusion

Aging is better understood as a set, like in mathematics, comprising various members - age-related diseases and conditions. The “set” is an abstraction, as aging is not a disease in itself, but a collection of diseases [28]. Detailing a quasi-program of aging is challenging without delving into the distinct pathological processes it encompasses. This is akin to describing a bouquet of flowers - while one can broadly call it beautiful and colorful, a detailed description necessitates a focus on individual flowers.

Aging can be viewed as a mathematical compilation of diseases and conditions. These “quasi-programs” represent developmental growth that wasn’t switched off, leading to hyperfunctions that damage organs and result in loss of function. Understanding aging, therefore, requires studying these early-life hyperfunctions. To improve longevity, one must concentrate on potential life-limiting diseases, designing appropriate doses and schedules of treatments like rapamycin, sometimes in combination with other drugs. This approach represents a blend of traditional medicine and geroscience, aptly termed as “geromedicine”.
